# Renal cell carcinoma and risk of second primary cancer: A Danish nationwide cohort study

**DOI:** 10.1002/cam4.7237

**Published:** 2024-06-13

**Authors:** Maria B. Bengtsen, Dóra K. Farkas, Henrik T. Sørensen, Mette Nørgaard

**Affiliations:** ^1^ Department of Clinical Epidemiology Aarhus University Hospital Aarhus Denmark; ^2^ Department of Clinical Medicine Aarhus University Aarhus Denmark

**Keywords:** cohort, kidney cancer, metachronous neoplasms, renal cell carcinoma, second primary cancer, second primary neoplasms

## Abstract

**Aim:**

To examine the risk of second primary cancer in patients with incident renal cell carcinoma (RCC).

**Methods:**

We identified all patients diagnosed with incident RCC during 1995–2019, using population‐based Danish medical registries. Patients were followed from the date of RCC diagnosis until any second primary cancer diagnosis, death, emigration, or December 31, 2019, whichever came first. We computed the absolute risk, standardized incidence ratio (SIR), and excess absolute risk of second primary cancer, with 95% confidence intervals (CIs), among patients with RCC compared to the general population.

**Results:**

The absolute 1‐ and 20‐year risks of any second primary cancer were 2.8% and 17.8%, respectively. Within 1 year after RCC diagnosis, we detected 20 excess cancer cases per 1000 person‐years (PY) (SIR, 2.3; 95% CI: 2.1–2.6). Moreover, we detected an additional four excess cancer cases per 1000 PY during 1 to <5 years of follow‐up (SIR, 1.3; 95% CI: 1.2–1.4), and 6 per 1000 PY beyond 5 years of follow‐up (SIR, 1.4; 95% CI: 1.3–1.5). The sustained elevated cancer risk beyond 1 year of follow‐up was mainly attributed to excess risk of lung and bladder cancer. The risk of second primary cancer was higher in 2006–2019 than in 1995–2005, but only during the first year of follow‐up.

**Conclusion:**

Patients with incident RCC have a sustained 40% elevated long‐term risk of second primary cancer, compared with the general population. This increased risk is mainly attributed to lung and bladder cancer.

## 1 INTRODUCTION

Renal cell carcinoma (RCC) is one of the 10 most frequently diagnosed cancers worldwide, with the highest incidence in Western countries.^1^ It is nearly twice as prevalent in men as women, and well‐established risk factors include smoking, obesity, and hypertension.^2^ The RCC incidence has been increasing over time in most countries, while mortality has stabilized or decreased since the 1990s.^1^ This pattern is attributed to several factors, including a rising prevalence of obesity and hypertension, as well as increased incidental detection of early‐stage RCC through more frequent abdominal imaging.^1^, ^3^ Moreover, prior to the introduction of targeted therapy, there was a dearth of effective systemic therapies for metastatic RCC and the prognosis was poor.^1^ However, since 2005, the introduction of several systemic treatments for metastatic RCC has improved patients' survival.^3^

Altogether, the increasing incidence and improved survival has resulted in a growing number of RCC survivors who are at risk of developing subsequent malignancies. Knowledge about the risk of subsequent cancers among RCC survivors is important for assessing their prognosis and need for long‐term follow‐up. Several potential mechanisms may contribute to their elevated risk of developing secondary malignancies, including shared risk factors, cancer treatments, and genetic predisposition.

Previous studies have examined the risk of second primary cancer among patients with RCC.^4^, ^5^, ^6^, ^7^, ^8^, ^9^, ^10^, ^11^ However, most were conducted before the introduction of systemic therapies for metastatic RCC,^4^, ^6^, ^7^, ^9^, ^10^, ^11^ and no data have been published beyond 2013. Therefore, we conducted a nationwide population‐based cohort study of primary RCC patients, to examine their long‐term risk of second primary cancers, with specific focus on temporality and the evaluation of relative, absolute, and excess risks.

## 2 MATERIALS AND METHODS

### 2.1 Setting and design

We conducted a nationwide cohort study in Denmark, which had a cumulative population of 7.7 million inhabitants during the study period.^12^ The Danish National Health Services provide tax‐supported healthcare to all residents, guaranteeing free and equal access to primary care and hospitals.^13^ Upon birth or immigration, all Danish residents are assigned a unique central personal registry number, enabling complete, and valid linkage of all registries at the individual level.^14^

### 2.2 Study population

From the Danish Cancer Registry (DCR),^15^ we identified all patients, aged 18 years or above, who were diagnosed with incident RCC between January 1, 1995 and December 31, 2019. The DCR contains prospectively collected data, including cancer stage, regarding all cancers diagnosed in Denmark since 1943.^15^ The DCR is routinely subjected to scheduled quality controls, and has an estimated 95%–98% completeness and accuracy rate of the recorded diagnoses.^15^ To restrict our cohort to patients with incident cancer, we excluded patients who had been diagnosed with other cancer (excluding non‐melanoma skin cancer) prior to their RCC diagnosis.

### 2.3 Outcome

We used the DCR to obtain information about second primary cancers diagnosed after incident RCC—including all cancer cases except non‐melanoma skin cancer and RCC. Table 2 shows how second primary cancers were categorized. Cancers occurring in less than five cases within the first year after RCC diagnosis were included in the main analysis, but not reported separately.

### 2.4 Comorbidity

From the Danish National Patient Registry (DNPR), we obtained information regarding nephrectomy and comorbidity associated with established acquired risk factors of RCC, including diabetes, hypertension, dyslipidaemia, obesity, and chronic obstructive pulmonary disease.^16^ The DNPR contains data about all inpatient hospital contacts in Denmark since 1977, and all contacts with emergency rooms and outpatient clinics since 1995. Data regarding subsequent systemic therapy and ablative therapy were obtained starting in 2002 and 2005, respectively, due to limited data availability from earlier time periods. Table S1 presents the relevant definitions and codes.

### 2.5 Statistical analysis

We characterized RCC patients according to median age, median follow‐up time, sex, age groups (18–49 years, 50–59 years, 60–69 years, 70+ years), calendar period of RCC diagnosis corresponding to the 2006 introduction of the first targeted therapies in Denmark (1995–2005, 2006–2019), RCC stage (localized, regional spread, distant metastasis, and unknown/missing), and presence of other comorbidity as listed in Table 1. Patients were followed from incident RCC diagnosis until occurrence of cancer, death, emigration, or December 31, 2019, whichever came first. We computed the absolute risk of cancer, treating death as a competing risk.^17^ As a measure of relative risk, we computed standardized incidence ratios (SIRs), as the ratio of the observed number of cancers in the RCC cohort and the expected number of cancers, based on national cancer incidence rates, according to sex, age (5‐year groups), and calendar year (5‐year groups). The excess absolute risk (EAR) was computed as the difference between the observed and expected number of cancers, divided by the follow‐up period. Confidence intervals (CIs) were computed assuming that the observed number of cancer cases followed a Poisson distribution. Exact confidence limits were calculated when there were fewer than 10 observed cancer cases; otherwise Byar's approximation was used. We estimated the risk of second primary cancer overall, as well as stratified by cancer site, calendar year group, sex, age group, and cancer stage.

**TABLE 1:** Characteristics of patients diagnosed with renal cell carcinoma in Denmark, 1995–2019.

	Women	Men	All patients
	5135 (100)	9414 (100)	14,549 (100)
Median follow‐up time, years (IQR)	2.3 (0.5–6.4)	2.2 (0.5–6.1)	2.2 (0.5–6.2)
Median age, years (IQR)	69 (59–77)	65 (57–73)	66 (58–74)
Age at RCC diagnosis			
18–49 years	471 (9)	1061 (11)	1532 (11)
50–59 years	896 (17)	2092 (22)	2988 (21)
60–69 years	1412 (28)	2942 (31)	4354 (30)
70+ years	2356 (46)	3319 (35)	5675 (39)
Calendar period			
1995–2005	1899 (37)	3033 (32)	4932 (34)
2006–2019	3236 (63)	6381 (68)	9617 (66)
RCC stage			
Localized	2782 (54)	5170 (55)	7952 (55)
Regional spread	248 (5)	401 (4)	649 (5)
Distant metastasis	1283 (25)	2395 (25)	3678 (25)
Unknown/missing	822 (16)	1448 (15)	2270 (16)
Histologic subtype			
Clear cell carcinoma	3857 (75)	7429 (79)	11,286 (78)
Papillary adenocarcinoma	179 (4)	557 (6)	736 (5)
Other RCC with grouped morphology	353 (7)	537 (6)	890 (6)
Other RCC without grouped morphology	746 (15)	891 (10)	1637 (11)
Treatment within first 6 months of RCC diagnosis			
Nephrectomy	2784 (54)	5303 (56)	8087 (56)
Systemic therapy^a^	450 (11)	1058 (14)	1508 (13)
Ablative therapy^b^	213 (6)	459 (7)	672 (7)
Other comorbidity			
Diabetes	493 (10)	986 (11)	1479 (10)
Hypertension	1285 (25)	2322 (25)	3607 (25)
Dyslipidaemia	357 (7)	873 (9)	1230 (9)
Obesity	441 (9)	428 (5)	869 (6)
Chronic obstructive pulmonary disease	540 (11)	827 (9)	1367 (9)

*Note*: Data are presented as number (%) unless otherwise specified.

Abbreviations: IQR, interquartile range; RCC, renal cell carcinoma.

^a^ Restricted to 2002 and later years due to limited data availability.

^b^ Restricted to 2005 and later years due to limited data availability.

Statistical analyses were performed using SAS statistical software package, version 9.4 (SAS Institute, Cary, NC). This study was reported to the Danish Data Protection Agency through registration at Aarhus University (record no. KEA‐2017‐36/812).

## 3 RESULTS

We identified 14,549 patients (65% men) diagnosed with incident RCC during 1995–2019 (Table 1). Of these, 7429 (79%) were clear cell carcinoma, 736 (5.1%) were papillary adenocarcinoma, and 2527 were (17.4%) other RCC or with undefined morphylogy. The median age was 66 (interquartile range [IQR], 58–74) and median follow‐up was 2.2 years (IQR, 0.5–6.2). At the time of RCC diagnosis, 7952 (55%) had localized RCC, 649 (5%) regional spread disease, 3678 (25%) distant metastatic disease, and 2270 (16%) unknown stage.

### 3.1 Risk of any cancer

During the first year of follow‐up, we observed 395 second primary cancers in our patient population, compared to 169 expected cancers, corresponding to 20 excess cancers per 1000 person‐years (PY) (SIR, 2.3) (Table 2). During 1 to <5 years and 5+ years of follow‐up, we detected an excess of 4 and 6 cancers per 1000 person‐years, respectively. The SIR values were highest within the first year of follow‐up. However, beyond 1 and 5 years of follow‐up, patients with primary RCC maintained a 1.3‐ to 1.4‐fold elevated risk of any cancer compared to the risk in the general population.

**TABLE 2:** Excess absolute risk and standardized incidence ratios of second primary cancer in 14,549 patients with incident renal cell carcinoma, stratified by cancer site.

Cancer site	Follow‐up								
	<1 year			1 to <5 years			5+ years		
	O/E	SIR (95% CI)	EAR per 1000 PY (95% CI)	O/E	SIR (95% CI)	EAR per 1000 PY (95% CI)	O/E	SIR (95% CI)	EAR per 1000 PY (95% CI)
Any cancer	395/169	2.3 (2.1–2.6)	20 (17–24)	526/413	1.3 (1.2–1.4)	4 (1–8)	549/404	1.4 (1.3–1.5)	6 (0–12)
Oral cavity and pharynx cancer	6/4	1.4 (0.5–3.1)	0 (0–1)	12/10	1.2 (0.6–2.1)	0 (−1–1)	10/9	1.1 (0.5–2.0)	0 (−1–1)
Respiratory cancer	65/27	2.4 (1.9–3.1)	3 (2–5)	106/64	1.6 (1.4–2.0)	2 (0–3)	76/61	1.2 (1.0–1.6)	1 (−2–3)
Lung, bronchi, and trachea	57/24	2.4 (1.8–3.1)	3 (2–4)	95/58	1.6 (1.3–2.0)	1 (0–3)	71/55	1.3 (1.0–1.6)	1 (−1–3)
Pleura	5/1	6.3 (2.0–14.6)	0 (0–1)	6/2	3.1 (1.1–6.7)	0 (0–1)	0/2	0.0 (0.0–0.0)	0 (0–0)
Gastrointestinal cancer	75/40	1.9 (1.5–2.3)	3 (1–5)	117/98	1.2 (1.0–1.4)	1 (−1–3)	125/98	1.3 (1.1–1.5)	1 (−2–4)
Stomach	9/3	2.7 (1.2–5.2)	1 (0–1)	7/8	0.9 (0.4–1.8)	0 (−1–0)	10/8	1.3 (0.6–2.4)	0 (−1–1)
Small intestine	5/1	8.2 (2.7–19.1)	0 (0–1)	NA	2.6 (0.7–6.6)	NA	6/2	3.7 (1.4–8.1)	0 (−0–1)
Colorectal cancer	33/25	1.3 (0.9–1.9)	1 (0–2)	69/60	1.1 (0.9–1.5)	0 (−1–2)	75/60	1.2 (1.0–1.6)	1 (−2–3)
Liver	9/2	4.0 (1.8–7.6)	1 (0–1)	14/6	2.5 (1.4–4.2)	0 (0–1)	NA	0.5 (0.1–1.6)	NA
Pancreas	13/5	2.6 (1.4–4.5)	1 (0–1)	13/12	1.1 (0.6–1.8)	0 (−1–1)	18/12	1.5 (0.9–2.4)	0 (−1–1)
Urological cancer	80/13	6.0 (4.7–7.4)	6 (4–7)	48/32	1.5 (1.1–2.0)	1 (−1–2)	45/32	1.4 (1.0–1.9)	1 (−1–2)
Renal pelvis	18/1	33.2 (19.7–52.4)	2 (1–2)	NA	3.1 (0.9–8.0)	NA	NA	2.5 (0.5–7.4)	NA
Urinary bladder	56/12	4.6 (3.4–5.9)	4 (3–5)	44/30	1.5 (1.1–2.0)	1 (−1–2)	41/29	1.4 (1.0–1.9)	1 (−1–2)
Female genital system cancer	8/6	1.4 (0.6–2.8)	1 (−1–2)	5/14	0.4 (0.1–0.9)	−1 (−2–1)	15/13	1.1 (0.6–1.9)	0 (−3–3)
Male genital system cancer	65/32	2.1 (1.6–2.6)	5 (2–7)	90/78	1.2 (0.9–1.4)	1 (−2–3)	83/77	1.1 (0.9–1.3)	0 (−3–4)
Melanoma skin cancer	11/7	1.5 (0.8–2.7)	0 (0–1)	27/19	1.5 (1.0–2.1)	0 (−1–1)	27/19	1.4 (0.9–2.1)	0 (−1–2)
Mesothelium and connective tissue cancer	6/1	6.2 (2.3–13.4)	0 (0–1)	NA	1.7 (0.5–4.3)	NA	NA	0.8 (0.1–3.1)	NA
Breast cancer	12/14	0.9 (0.4–1.5)	0 (−1–1)	39/34	1.1 (0.8–1.6)	0 (−1–1)	41/33	1.2 (0.9–1.7)	0 (−1–2)
Brain, eye, and orbit cancer	21/6	3.4 (2.1–5.2)	1 (1–2)	23/15	1.5 (1.0–2.3)	0 (0–1)	27/15	1.8 (1.2–2.6)	1 (−1–2)
Membrane of the brain and spinal meninx	6/2	3.6 (1.3–7.9)	0 (0–1)	NA	0.7 (0.1–2.1)	NA	10/4	2.3 (1.1–4.1)	0 (−0–1)
Brain	11/3	3.3 (1.7–6.0)	1 (0–1)	16/8	2.0 (1.2–3.3)	0 (0–1)	12/8	1.6 (0.8–2.8)	0 (−1–1)
Endocrine system cancer	6/1	8.4 (3.1–18.2)	0 (0–1)	9/2	5.0 (2.3–9.5)	0 (0–1)	NA	1.2 (0.1–4.2)	NA
Lymphatic cancer	26/8	3.1 (2.0–4.5)	2 (1–2)	26/21	1.2 (0.8–1.8)	0 (−1–1)	19/21	0.9 (0.5–1.4)	0 (−1–1)
Non‐Hodgkin malignant lymphoma	16/6	2.8 (1.6–4.5)	1 (0–2)	17/14	1.2 (0.7–1.9)	0 (−1–1)	12/15	0.8 (0.4–1.4)	0 (−1–1)
Multiple myeloma and other plasma cell neoplasms	8/2	3.5 (1.5–6.9)	1 (0–1)	6/6	1.1 (0.4–2.3)	0 (0–0)	5/6	0.9 (0.3–2.0)	0 (−1–1)
Hematopoietic cancer	10/5	2.2 (1.0–4.0)	0 (0–1)	16/11	1.4 (0.8–2.3)	0 (0–1)	17/11	1.5 (0.9–2.4)	0 (−1–1)
Lymphoid leukemia	6/3	2.1 (0.8–4.7)	0 (0–1)	7/7	1.0 (0.4–2.1)	0 (0–0)	11/7	1.6 (0.8–2.9)	0 (−1–1)

*Note*: Cancers occurring in less than five cases within 1 year of RCC diagnosis were included in the main analysis, but not reported separately (i.e., are reported as NA).

Abbreviations: EAR, excess absolute risk; NA, not applicable; O/E, observed/expected; PY, person‐years; SIR, standardized incidence ratio.

### 3.2 Risk by cancer site

During the first year of follow‐up, we observed an increased risk of nearly all investigated cancers (Table 2). The highest EAR values were observed for urological cancers (EAR, 6/1000 PY), mainly driven by bladder cancer (EAR, 4/1000 PY); male genital cancer (EAR, 5/1000 PY), driven by prostate cancer (5/1000 PY); respiratory cancer (EAR, 3/1000 PY), driven by lung cancer (EAR, 3/1000 PY); and gastrointestinal cancer (EAR, 3/1000 PY). During 1 to <5 years and 5+ years of follow‐up, the EAR was rather stable at 1 excess cancer per 1000 PY for gastrointestinal cancers and urological cancer (driven by bladder cancer), while the EAR decreased from 2 to 1 for respiratory cancer and from 1 to 0 for male genital cancer.

The SIR values were highest within the first year of follow‐up, but remained elevated for most cancers during 1 to <5 and beyond 5 years of follow‐up. Beyond 1 and 5 years of follow‐up, patients with primary RCC had a 1.2‐ to 1.6‐fold risk of respiratory, gastrointestinal, urological, hematopoietic, and melanoma skin cancer.

After incident RCC, the 1‐year and 20‐year absolute risks of cancer, respectively, were 0.6% and 1.8% for urological cancer (0.4% and 1.6% for bladder cancer), 0.7% and 4.4% for male genital cancer (0.7% and 4.3% for prostate cancer), 0.5% and 2.9% for respiratory cancer (0.4% and 2.6% for lung cancer), and 0.5% and 4.0% for gastrointestinal cancer. After 1 year of follow‐up, the absolute risks of cancer at other sites were 0.2% or less.

### 3.3 Risk by calendar year

The SIR and EAR values during the first year of follow‐up were higher in 2006–2019, compared to in 1995–2005 (Table 3). Beyond 1 year of follow‐up, the impact of calendar year group on the risk of subsequent cancer attenuated.

**TABLE 3:** Excess absolute risk and standardized incidence ratios of second primary cancer in 14,549 patients with incident renal cell carcinoma, stratified by calendar year group.

	Follow‐up								
	<1 year			1 to <5 years			5+ years		
	O/E	SIR (95% CI)	EAR per 1000 PY	O/E	SIR (95% CI)	EAR per 1000 PY	O/E	SIR (95% CI)	EAR per 1000 PY
Any cancer									
1995–2005	79/48	1.7 (1.3–2.1)	9 (4–14)	146/121	1.2 (1.0–1.4)	3 (−4–9)	347/240	1.4 (1.3–1.6)	8 (−2–18)
2006–2019	316/122	2.6 (2.3–2.9)	25 (20–30)	380/292	1.3 (1.2–1.4)	5 (0–10)	202/164	1.2 (1.1–1.4)	4 (−3–11)
Respiratory cancer									
1995–2005	16/8	1.9 (1.1–3.1)	2 (0–5)	28/21	1.4 (0.9–2.0)	1 (−2–4)	53/37	1.4 (1.1–1.9)	1 (−3–5)
2006–2019	49/18	2.7 (2.0–3.5)	4 (2–6)	78/44	1.8 (1.4–2.2)	2 (0–4)	23/24	0.9 (0.6–1.4)	0 (−3–3)
Gastrointestinal cancer									
1995–2005	14/12	1.2 (0.7–2.0)	1 (−2–3)	36/29	1.2 (0.9–1.7)	1 (−2–4)	80/58	1.4 (1.1–1.7)	2 (−3–6)
2006–2019	61/29	2.1 (1.6–2.7)	4 (2–6)	81/69	1.2 (0.9–1.5)	1 (−2–3)	45/40	1.1 (0.8–1.5)	1 (−3–4)
Urological cancer									
1995–2005	12/4	2.9 (1.5–5.1)	2 (0–4)	10/10	1.0 (0.5–1.8)	0 (−2–2)	26/19	1.4 (0.9–2.0)	1 (−2–3)
2006–2019	68/9	7.3 (5.7–9.3)	8 (6–10)	38/22	1.7 (1.2–2.3)	1 (−1–2)	19/13	1.5 (0.9–2.3)	1 (−1–3)
Male genital cancer									
1995–2005	14/7	2.0 (1.1–3.4)	3 (0–7)	27/21	1.3 (0.9–1.9)	1 (−3–6)	53/45	1.2 (0.9–1.5)	1 (−6–8)
2006–2019	51/25	2.1 (1.6–2.7)	5 (2–8)	63/57	1.1 (0.9–1.4)	1 (−2–4)	30/32	1.0 (0.6–1.4)	0 (−5–4)

Abbreviations: EAR, excess absolute risk; O/E, observed/expected; PY, person‐years; SIR, standardized incidence ratio.

### 3.4 Risk by sex, age, and RCC stage

Analyses stratified by age revealed that the SIRs tended to be attenuated with increasing age within the first year of follow‐up, for all investigated cancer groups—except male genital cancers, which were unaffected by age (Table 4). In contrast, EAR was only affected by age in male genital cancer, where we observed a higher EAR among men aged 70+ (9/1000 PY) compared to in men aged 18–69 years (2/1000 PY). Sex did not impact the risk of second primary cancer with regards to respiratory cancer or gastrointestinal cancers. On the other hand, within the first year of follow‐up, men had a higher EAR for urological cancer than women (8/1000 and 3/1000 PY, respectively). The effects of sex and age tended to dilute with increasing follow‐up period.

**TABLE 4:** Excess absolute risk and standardized incidence ratios of second primary cancer in 14,549 patients with incident renal cell carcinoma, stratified by sex, age group, and renal cell carcinoma stage.

	<1 year			1 to <5 years			5+ years		
	O/E	SIR (95% CI)	EAR per 1000 PY (95% CI)	O/E	SIR (95% CI)	EAR per 1000 PY (95% CI)	O/E	SIR (95% CI)	EAR per 1000 PY (95% CI)
Any cancer									
Total	395/169	2.3 (2.1–2.6)	20 (17–24)	526/413	1.3 (1.2–1.4)	4 (1–8)	549/404	1.4 (1.3–1.5)	6 (0–12)
Female	106/52	2.0 (1.7–2.5)	14 (8–19)	150/130	1.2 (1.0–1.4)	2 (−4–8)	186/130	1.4 (1.2–1.7)	7 (−3–16)
Male	289/117	2.5 (2.2–2.8)	24 (19–28)	376/283	1.3 (1.2–1.5)	6 (1–10)	363/274	1.3 (1.2–1.5)	6 (−1–14)
Age									
18–69	223/74	3.0 (2.6–3.4)	20 (16–25)	300/217	1.4 (1.2–1.6)	4 (0–9)	414/293	1.4 (1.3–1.6)	7 (−0–13)
70+	172/95	1.8 (1.6–2.1)	20 (13–26)	226/196	1.2 (1.0–1.3)	4 (−4–12)	135/111	1.2 (1.0–1.4)	6 (−8–19)
RCC stage									
Localized	231/106	2.2 (1.9–2.5)	17 (13–22)	405/306	1.3 (1.2–1.5)	5 (1–10)	420/318	1.3 (1.2–1.5)	6 (−1–12)
Non‐localized	90/37	2.4 (1.9–3.0)	21 (15–28)	44/44	1.0 (0.7–1.3)	−0 (−9–9)	45/24	1.9 (1.4–2.5)	15 (−8–37)
Unknown	74/26	2.9 (2.2–3.6)	30 (20–41)	77/62	1.2 (1.0–1.5)	4 (−7–14)	84/62	1.4 (1.1–1.7)	7 (−9–22)
Respiratory cancer									
Total	65/27	2.4 (1.9–3.1)	3 (2–5)	106/64	1.6 (1.4–2.0)	2 (0–3)	76/61	1.2 (1.0–1.6)	1 (−2–3)
Female	23/8	3.0 (1.9–4.5)	4 (1–6)	37/20	1.9 (1.3–2.6)	2 (−1–4)	29/19	1.5 (1.0–2.1)	1 (−3–5)
Male	42/19	2.2 (1.6–3.0)	3 (1–5)	69/45	1.5 (1.2–2.0)	1 (−1–3)	47/42	1.1 (0.8–1.5)	0 (−3–3)
Age									
18–69	39/11	3.4 (2.5–4.7)	4 (2–6)	65/33	2.0 (1.5–2.5)	2 (0–4)	56/45	1.2 (0.9–1.6)	1 (−2–3)
70+	26/15	1.7 (1.1–2.5)	3 (0–5)	41/31	1.3 (0.9–1.8)	1 (−2–5)	20/16	1.2 (0.8–1.9)	1 (−4–6)
RCC stage									
Localized	39/17	2.4 (1.7–3.2)	3 (1–5)	89/48	1.9 (1.5–2.3)	2 (0–4)	63/48	1.3 (1.0–1.7)	1 (−2–3)
Non‐localized	16/6	2.7 (1.5–4.3)	4 (1–7)	5/7	0.7 (0.2–1.7)	−1 (−4–3)	7/4	1.9 (0.8–4.0)	2 (−7–11)
Unknown	10/4	2.5 (1.2–4.6)	4 (0–8)	12/10	1.2 (0.6–2.2)	1 (−4–5)	6/9	0.6 (0.2–1.4)	−1 (−6–4)
Gastrointestinal cancer									
Total	75/40	1.9 (1.5–2.3)	3 (1–5)	117/98	1.2 (1.0–1.4)	1 (−1–3)	125/98	1.3 (1.1–1.5)	1 (−2–4)
Female	20/12	1.7 (1.1–2.7)	2 (0–5)	25/29	0.9 (0.6–1.3)	0 (−3–2)	37/30	1.2 (0.9–1.7)	1 (−3–5)
Male	55/29	1.9 (1.5–2.5)	4 (1–6)	92/69	1.3 (1.1–1.6)	1 (−1–4)	88/68	1.3 (1.0–1.6)	1 (−2–5)
Age									
18–69	40/16	2.5 (1.8–3.4)	3 (1–5)	68/48	1.4 (1.1–1.8)	1 (−1–3)	85/68	1.3 (1.0–1.6)	1 (−2–4)
70+	35/24	1.4 (1.0–2.0)	3 (−0–6)	49/50	1.0 (0.7–1.3)	0 (−4–4)	40/30	1.3 (1.0–1.8)	2 (−5–10)
RCC stage									
Localized	39/25	1.6 (1.1–2.1)	2 (−0–4)	88/73	1.2 (1.0–1.5)	1 (−1–3)	102/77	1.3 (1.1–1.6)	1 (−2–5)
Non‐localized	13/9	1.5 (0.8–2.5)	2 (−1–4)	8/11	0.8 (0.3–1.5)	−1 (−5–3)	9/6	1.6 (0.7–3.0)	2 (−8–13)
Unknown	23/6	3.7 (2.3–5.5)	11 (5–16)	21/15	1.4 (0.9–2.1)	2 (−4–7)	14/15	0.9 (0.5–1.6)	0 (−7–7)
Urological cancer									
Total	80/13	6.0 (4.7–7.4)	6 (4–7)	48/32	1.5 (1.1–2.0)	1 (−1–2)	45/32	1.4 (1.0–1.9)	1 (−1–2)
Female	13/2	6.4 (3.4–11.0)	3 (1–4)	7/5	1.4 (0.6–2.8)	0 (−1–1)	5/5	1.0 (0.3–2.3)	0 (−2–2)
Male	67/11	5.9 (4.6–7.5)	8 (6–10)	41/27	1.5 (1.1–2.0)	1 (−1–2)	40/27	1.5 (1.1–2.0)	1 (−2–3)
Age									
18–69	47/5	9.4 (6.9–12.5)	6 (4–8)	18/15	1.2 (0.7–1.9)	0 (−1–1)	31/22	1.4 (1.0–2.0)	1 (−1–2)
70+	33/8	3.9 (2.7–5.5)	6 (4–9)	30/17	1.7 (1.2–2.5)	2 (−1–4)	14/10	1.4 (0.8–2.3)	1 (−3–5)
RCC stage									
Localized	46/8	5.6 (4.1–7.5)	5 (3–7)	39/24	1.6 (1.2–2.2)	1 (−1–2)	31/25	1.2 (0.8–1.8)	0 (−2–2)
Non‐localized	19/3	6.2 (3.7–9.7)	7 (4–9)	NA	0.6 (0.1–2.0)	NA	NA	0.5 (0.0–3.0)	NA
Unknown	15/2	7.1 (4.0–11.7)	8 (4–12)	NA	1.4 (0.6–2.9)	NA	NA	2.6 (1.4–4.4)	NA
Male genital cancer									
Total	65/32	2.1 (1.6–2.6)	5 (2–7)	90/78	1.2 (0.9–1.4)	1 (−2–3)	83/77	1.1 (0.9–1.3)	0 (−3–4)
Age									
18–69	26/14	1.9 (1.3–2.8)	2 (0–5)	51/42	1.2 (0.9–1.6)	1 (−2–3)	67/59	1.1 (0.9–1.5)	1 (−4–5)
70+	39/18	2.2 (1.5–3.0)	9 (4–15)	39/36	1.1 (0.8–1.5)	1 (−5–7)	16/18	0.9 (0.5–1.4)	−1 (−11–9)
RCC stage									
Localized	44/20	2.2 (1.6–3.0)	5 (2–8)	65/57	1.1 (0.9–1.5)	1 (−2–4)	66/60	1.1 (0.9–1.4)	1 (−4–5)
Non‐localized	11/7	1.6 (0.8–2.9)	3 (−1–6)	10/9	1.2 (0.6–2.1)	1 (−5–7)	NA	0.6 (0.1–1.9)	NA
Unknown	10/5	2.0 (1.0–3.8)	5 (−1–11)	15/12	1.3 (0.7–2.1)	1 (−6–9)	NA	1.1 (0.6–1.9)	NA

*Note*: Cancers occurring in less than five cases within the first year of RCC diagnosis were included in the main analysis, but not reported separately (i.e., are reported as NA).

Abbreviations: EAR, excess absolute risk; NA, not applicable; O/E, observed/expected; PY, person‐years; RCC, renal cell carcinoma; SIR, standardized incidence ratio.

We observed no substantial difference in cancer risk according to whether RCC has been localized versus non‐localized disease. Patients with unknown RCC stage exhibited a slightly higher risk of gastrointestinal cancer within the first year after RCC diagnosis.

## 4 DISCUSSION

In this population‐based study, we found that patients with RCC had a higher risk of second primary cancer compared with the cancer risk in the general population, particularly during the most recent calendar year period. Within1 year after RCC diagnosis, patients exhibited an elevated risk of almost all investigated cancers. Although the SIR values were particularly high during the first year following RCC diagnosis, RCC patients maintained a 30%–40% elevated risk of cancer throughout follow‐up, compared to the general population. The sustained elevated risk of cancer was mainly attributed to excess risk of bladder and lung cancers.

Previous studies have yielded inconsistent results regarding the relationship between RCC and lung cancer, with some studies supporting an association,^4^, ^5^, ^8^, ^11^ while others have not.^6^, ^7^, ^9^, ^10^ There are also discrepancies in the existing evidence of association between RCC and colorectal cancer, with some studies supporting an association^4^, ^5^, ^11^ and others not.^6^, ^7^, ^8^, ^9^, ^10^ On the other hand, all previous studies show that RCC is associated with bladder cancer.^4^, ^5^, ^6^, ^7^, ^8^, ^9^, ^10^, ^11^ It is possible that differences between countries may be related to differences in genetic background, ethnic group, and lifestyle and environmental factors.

Only two previous studies have examined the risk of second cancer among RCC patients according to follow‐up time.^5^, ^11^ Both were Swedish population‐based studies, and both reported persistently elevated SIRs for most cancers, even beyond 10 years of follow‐up,^5^, ^11^ consistent with our present findings. Prior studies of the association between RCC and second primary cancers have primarily focused on the SIRs.^4^, ^5^, ^6^, ^7^, ^8^, ^9^, ^10^, ^11^ As a measure of relative risk, an SIR can indicate shared aetiologic factors, but does not reflect absolute risk estimates. Therefore, an exclusive focus on SIRs can convolute the clinical interpretations of the findings. This was highlighted by our finding that the excess risk of second primary cancer among RCC patients was mainly driven by bladder cancer and lung cancer—despite the fact that these cancers did not have the highest SIRs. Therefore, our present data expand on the previous literature, by demonstrating that although RCC was associated with most cancers, the excess long‐term cancer risk in patients with RCC was mainly driven by a sustained elevated risk of bladder and lung cancers.

The mechanisms through which RCC is associated with other cancers may be complex and multifactorial. These associations may result from enhanced diagnostic effort, treatment effects, and shared risk factors. Stratification by follow‐up may highlight the distinctions between these factors. For example, the elevated cancer risk observed in RCC patients within the first years of follow‐up may be largely due to enhanced diagnostic effort, such as increased use of surveillance imaging, leading to the detection of tumors that might not otherwise have been detected or clinically manifested. In our study, the SIRs remained relatively stable or decreased beyond 1 year of follow‐up. We observed the highest excess risk for bladder and lung cancers, suggesting that shared risk factors play a significant role in the association between RCC and second primary cancers. Cigarette smoking increases the RCC risk by 50% in men and 20% in women, compared to non‐smokers.^18^ Notably, cigarette smoking is also the leading behavioral risk factor for cancer worldwide,^19^ and the number one risk factor for lung and bladder cancer, linked to over 80% of all lung cancer cases^20^, ^21^ and approximately 50% of all bladder cancer cases.^22^ The association between gastrointestinal cancers and RCC might also be partly explained by shared risk factors, such as cigarette smoking, high body mass index, and hypertension.^18^, ^23^, ^24^ Hereditary renal cancers are estimated to account for 5%–8% of all renal tumors,^25^ and hereditary syndromes, such as Von Hippel Lindau and Cowden syndrome, can present with RCC and are also associated with other cancer types. This association could also contribute to the observed elevated cancer risk among RCC patients.^25^, ^26^ In recent decades, the prevalence rates of several shared risk factors for RCC and other cancers have substantially increased, including diabetes, obesity, and hypertension.^1^, ^3^ In our present study, we found that calendar year group only affected the risk of second primary cancers during the first year of follow‐up. This suggests that the elevated risk of second primary cancer during the most recent time period can be primarily explained by enhanced diagnostic effort or improved and readily available imaging modalities, rather than changes in RCC treatment or a more pronounced increase of risk factors among RCC patients compared with the general population.

Several strengths and limitations should be considered when interpreting our results. An important strength of our study compared with previous investigations, include the addition of excess and absolute risk estimates, and the examination of the risk of second primary cancers according to follow‐up time, baseline characteristics, and calendar period. Moreover, selection bias was largely eliminated by the population‐based design in a country with tax‐supported universal healthcare access, ensuring that all citizens had free access to primary and secondary care, and virtually complete follow‐up. Due to the completeness of the DCR, we expect that we captured virtually all cancer diagnoses.^15^ Misclassification of second primary cancers as metastases could have potentially led to underestimation of the risk of second primary cancer among RCC patients. However, given the high validity of primary cancer diagnoses in the DCR,^15^ we do not expect that misclassification had a major impact on findings of our study. Another limitation is that we did not have information on cigarette smoking and other life style factors, and therefore could not estimate the risk of second primary cancer according to these. Hence, the degree to which RCC patients without a history of smoking are susceptible to elevated risks of these cancers remains to be elucidated. For cancers other than lung and bladder cancer, the excess risk was less than 1 per 1000 person years after 1 year of follow‐up, suggesting that surveillance for these cancer types is unlikely to be profitable in RCC patients. Most cases of RCC occur sporadically, while hereditary renal cancers have been estimated to account for 5%–8% of all renal tumors. Whether a family history of RCC warrants surveillance/screening of RCC was beyond the scope of this study.

## 5 CONCLUSIONS

Beyond 5 years of follow‐up, patients with RCC sustained a persistent 40% elevated long‐term risk of a second primary cancer compared with the general population, mainly attributed to smoking‐related cancer located in the lungs and bladder.

## AUTHOR CONTRIBUTIONS

**Maria B. Bengtsen:** Conceptualization (equal); investigation (lead); methodology (equal); project administration (lead); writing – original draft (lead); writing – review and editing (equal). **Dóra K. Farkas:** Conceptualization (equal); formal analysis (lead); investigation (equal); methodology (equal); supervision (equal); writing – original draft (supporting); writing – review and editing (equal). **Henrik T. Sørensen:** Conceptualization (equal); data curation (lead); investigation (equal); methodology (equal); supervision (equal); writing – original draft (supporting); writing – review and editing (equal). **Mette Nørgaard:** Conceptualization (equal); investigation (equal); methodology (equal); project administration (supporting); supervision (lead); writing – original draft (supporting); writing – review and editing (lead).

## FUNDING INFORMATION

This research did not receive any specific grant from funding agencies in the public, commercial, or not‐for‐profit sectors.

## CONFLICT OF INTEREST STATEMENT

The authors declare no conflict of interest.

## ETHICS STATEMENT

The study was approved by the Danish Data Protection Agency through registration at Aarhus University (record number KEA‐2017‐36/812). In accordance with Danish regulations governing analysis of registry data, no Ethics Committee approval was required.

## Supporting information


**Table S1.** International Classification of Diseases (ICD) codes.

## Data Availability

The data used in the current study are not freely available owing to national regulations. Interested researchers may apply for data access through the Research Service at the Danish Health Data Authority.
