# Supplementary material for: Renal cell carcinoma and risk of second primary cancer: A Danish nationwide cohort study
Source: Cancer Med. 2024 Jun 13;13(11):e7237. doi: 10.1002/cam4.7237 (PMC11176587; doi:10.1002/cam4.7237)
Supplement: Supplementary file 1 — Table S1. International Classification of Diseases (ICD) codes. [file CAM4-13-e7237-s001.docx]

**Supplementary Table 1**. International Classification of Diseases (ICD) codes.

|  | ICD-8 | ICD-10 | C64 |
| --- | --- | --- | --- |
| **Exposure** |  |  |  |
| Renal cell carcinoma |  | C64 |  |
| Clear cell adenocarcinomas |  |  | 83103, 83106, 83109 83123, 83126, 83129 |
| Papillary adenocarcinomas |  |  | 80503, 82603, 82903, 84503, 82609 |
| Other |  |  | 80103, 81403, 82303, 82703, 83173, 83183 82113 |
| **Outcome** |  |  |  |
| **Any cancer*** |  | C00-C96, B210, D090-091, D095-096, D301-309, 411-419,  D32-33, D352-354, D42-43, D443-445 |  |
| **Oral cavity and pharynx** |  | C00-C14 |  |
| **Respiratory system** |  | C30-C39, C450 |  |
| Lung, bronchi and trachea |  | C33-C34 |  |
| Pleura |  | C384, C450 |  |
| **Gastrointestinal cancer** |  | C15-26 |  |
| Stomach |  | C16 |  |
| Small intestine |  | C17 |  |
| Colorectal cancer |  | C18-20 |  |
| Liver |  | C22 |  |
| Pancreas |  | C25 |  |
| **Urological cancer** |  | C64-68, D090-091, D095-096, D301-309, D411-419** |  |
| Renal pelvis |  | C65, D301, D411** |  |
| Urinary bladder |  | C65, D090, D095, D303, D414** |  |
| **Female genital system** |  | C51-58 |  |
| **Male genital system** |  | C60-63 |  |
| Prostate |  | C61 |  |
| **Skin** |  | C43-44 |  |
| Non-melanoma skin cancer |  | C43 |  |
| Malignant melanoma |  | C44 |  |
| **Mesothelium and connective tissue** |  | C451-459, C46-49, B210 |  |
| **Breast** |  | C50 |  |
| **Brain, eye and orbit** |  | C69-72, C751-753, D32-33, D352-354, D42-43, D443-445 |  |
| Membrane of the brain and spinal meninx |  | C70, D32, D42 |  |
| Brain |  | C71, C751-753, D330-332, D352-354, D430-432, D443-445 |  |
| **Endocrine system** |  | C73-74, C750, C754-759 |  |
| **Lymphatic cancer** |  | C81-90 |  |
| Non-Hodgkin malignant lymphoma |  | C82-86, C90 |  |
| Multiple myeloma and other plasma cell neoplasms |  |  |  |
| **Hematopoietic cancer** |  | C91-96 |  |
| Lymphoid leukaemia |  | C91 |  |
| **Comorbidity and treatment within the six months of follow-up** | | |  |
| Diabetes | 249, 250 | E10, E11 |  |
| Chronic pulmonary disease | 490-493; 515-518 | J40-J47; J60-J67; J68.4; J70.1; J70.3; |  |
| Hypertension | 400-404 | I10-I15 |  |
| Metabolic syndrome |  | DE888C |  |
| Dyslipidemia | 272.00, 279.00, 279.01 | E78 |  |
| Obesity | 277 | E66 |  |
| Nephrectomy | 503-505 | KKAC, KKAD00, KKAD01, KKAD10, KKAD11 |  |
| Ablative therapies (data available since 2005) |  | KTKA30 |  |
| Systemic therapies (data available since 2002) |  | BWHA4, BOHJ1, BOHJ2, BWHB1, BWHB2 |  |
| * Except non-melanoma skin cancer and renal cell carcinoma | | |  |
| ** Furthermore, the D-codes are delimited by the morphologies 812-813. | |  |  |
